# Competitive Asymmetries in the Use of Supplementary Food by the Endangered Iberian Lynx *(Lynx pardinus)*

**DOI:** 10.1371/journal.pone.0007610

**Published:** 2009-10-28

**Authors:** José V. López-Bao, Alejandro Rodríguez, Francisco Palomares

**Affiliations:** Department of Conservation Biology, Estación Biológica de Doñana (CSIC), Seville, Spain; University of Pretoria, South Africa

## Abstract

**Background:**

As a conservation tool, supplementary feeding programs may be directed to specific individuals or sectors of the target population whose productivity or survival is thought to be limited by food scarcity. However, the use of supplemental food by different sex and age classes has received little attention. We studied individual variation in the access of the endangered Iberian lynx (*Lynx pardinus*) to supplementary food.

**Methodology/Principal Findings:**

From 5349 pictures taken with automatic cameras placed in 25 feeding stations, we identified 28 individuals whose sex and age were known. All individuals known to live in areas subjected to supplementation regularly visited feeding stations. Food consumption was not proportional to expected variations in energy demand within sex and age classes. Food consumption by males was higher than by females, and increased with age, in agreement with a despotic distribution. Food consumption also increased with lynx body mass, and this pattern held for individuals sharing the same breeding territories. The access of inferior competitors increased with the number of feeding stations available within lynx territories.

**Conclusions/Significance:**

All lynx exposed to food supplementation made a regular use of extra food but individuals predicted to be competitively dominant visited stations more frequently than subordinates of the same breeding territory. Our results suggest that insufficient provision of supplementary food could restrict the access of juveniles, or even adult females, to feeding stations. Limited consumption by these target individuals may compromise the efficiency of the supplementary feeding programme at the population level, in endangered species that, as the Iberian lynx, exhibit marked sexual dimorphism in body size.

## Introduction

Food supplementation experiments have long been used to explore whether predictors of fitness such as condition [1] and productivity [2], or population parameters such as survival [3] and recruitment [4], depend upon food availability. To a lesser extent, food supplementation has been employed for a number of applied purposes, including prevention of crop damage by wildlife [5], retention of translocated animals [6], or conservation of endangered populations [7], [8]. In a conservation context, supplementation is often targeted at specific sectors of a target population (i.e. sex and age classes) during periods when food limitation is thought to be critical [9].

The endangered Iberian lynx *(Lynx pardinus)* feeds almost exclusively upon European rabbits (*Oryctolagus cuniculus*; [10]), and rabbit scarcity has been identified as a major factor of lynx decline [11]. Even in protected areas inhabited by lynx, most sites do not reach the threshold rabbit density that allows lynx reproduction [12], [13] or settlement, which reduces the *per capita* productivity, extends the duration of natal dispersal, increases subadult mortality rates, and reduces recruitment [14], [15].

This situation prompted a rabbit supplementation programme [16] whose major aims included improving reproductive rates of Iberian lynx within existing territories as well as creating new ones by promoting disperser settlement. By enhancing territory quality, supplementary food should increase body condition and productivity of adult females, thus improving the reproductive output of the population. Food supplementation should also transform empty areas into potential territories, offering more opportunities for subadults to settle down, and to do it earlier, thus reducing dispersal time and associated high risk of mortality [14]. Since female settlement precedes male arrival and subsequent breeding, the supplementation programme implicitly identifies adult and juvenile females as target individuals.

The overall biomass of supplemented rabbits taken by lynx has been estimated to be twice that required to satisfy the energy requirements of the population fraction exposed to this resource [16], according to a model of metabolic cost [17]. However, it is unknown whether supplementary food is consumed evenly by different sex and age classes. Here we test two hypotheses regarding alternative mechanisms by which some sex and age classes could make a disproportionate use of supplementary food.

The demand hypothesis (DH) states that food intake should be proportional to variations in energetic requirements, so that each sex and age class would compensate their energy deficit by consuming supplemented rabbits. Adult females may require more food than adult males since the energy cost of reproduction for female mammals (gestation, lactation, offspring rearing) is higher than that for males (mate acquisition and resource defence; [18]). On the other hand, juveniles able to forage on their own may be less efficient hunters than adults [19] and may need comparatively more accessible, supplementary food than adults in a context of low abundance of wild prey. The energy demand of adult males would be highest prior and during the mating season, when they have to compete for access to resident females [17].

Another type of asymmetrical consumption is expected when food is clumped and predictable [20]–[22], two features that adequately describe how supplemented rabbits were delivered to lynx [16]. The despotic distribution hypothesis (DDH) states that conspecifics differ in competitive ability, and best competitors consume a large share of available food [23]. In most vertebrates, competitive ability increases with age and body size [24]. A clear hierarchy in the access to food has been documented between sex and age classes in species exhibiting sexual dimorphism in body size [20], [21]. Therefore, these classes may indicate the position of individuals in a competitive hierarchy. Since male Iberian lynx are heavier than females [25], and adults are heavier than juveniles of the same sex [25], [26], intraspecific dominance in food use of adults over juveniles, and of males over females, is expected under this hypothesis.

In this study we first test the null hypothesis that individual Iberian lynx use feeding stations and consume supplementary food irrespective of their sex, age and condition. Secondly we examine whether the DH or the DDH describe the pattern of food consumption by sex and age classes better than the null hypothesis. The DH predicts that 1) during the breeding season, resident females will use more supplementary food than at other times of the year; 2) a few weeks before and during the mating season, adult males will consume more food than outside the mating season; 3) breeding adult females will use more food than non-breeding adult females; and 4) young independent of their mothers will need and consume more supplementary food than either adult females outside the breeding season or adult males outside the mating season. The DDH predicts that 1) supplementary food will be consumed unequally according to the following hierarchy given by reproductive status and body size: adult male>adult female>subadult male>subadult female>juvenile male>juvenile female; 2) differences in consumption between sex and age classes will be reduced by increasing the amount and spatial dispersion of supplementary food. Finally, we discuss the implications of our results for the conservation of species that, as the Iberian lynx, exhibit a marked dimorphism in body size.

## Materials and Methods

### Ethics statement

This research complied with the norms of the Spanish Animal Protection Regulation, RD1201/2005, about protection of animals used in scientific research, which conforms to European Union Regulation 2003/65. Methods of capture and handling of wild lynx were specifically approved by the competent administration (Regional Government of Andalusia and the Doñana National Park) under permit No. RS-2093/04; whereas the provision of domestic rabbits was approved by the Regional Government of Andalusia through the implementation of the conservation project LIFE-02NAT/8609.

### Study area

**Figure 1:**
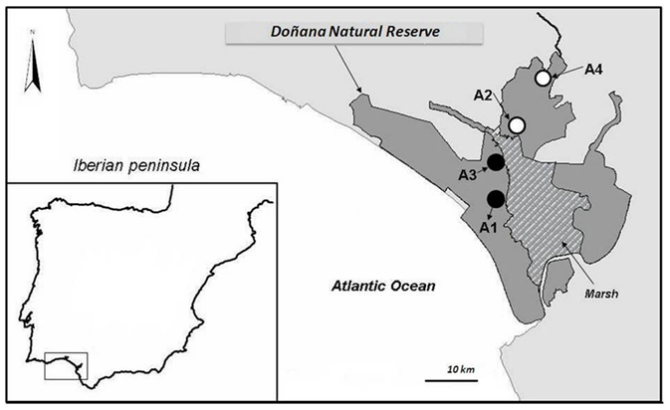
Location of the four lynx areas (circles) with resident lynx exposed to supplementary food within the Doñana region. Shaded area: protected area. Open circles: high lynx density (37.5–50 ind/100 km^2^). Black circles: low lynx density (7.3–15.8 ind/100 km^2^).

The study was carried out in the Doñana region, SW Spain (37°10′ N, 6°23′ W; 870 km^2^; Fig. 1). We studied the use of supplementary food by individual lynx in four lynx subpopulations inhabiting flat areas (A1–A4, 93 km^2^, Fig. 1), vegetated with scrubland (mainly *Halimium halimifolium*, *Ulex* spp., *Erica* spp. and *Pistacia lentiscus*), that differ in rabbit density and, hence, lynx density [27].

### Use of supplementary food by lynx

From 2002 to 2007, we provided alive domestic rabbits in 4×4 m enclosures (feeding stations, FS; n = 27; Table 1), that were evenly distributed with a density of 0.44 stations/km^2^ [16]. At least 1 rabbit (range: 1–7) was available per feeding station during 82% of days [16]. Iberian lynx entered easily feeding stations, used regularly the supplemental food, and consumed almost all the extra food in some frequently visited stations [16]. Supplementation with domestic rabbits was continuous throughout the year, and FS were usually checked every other day which guaranteed a predictable and abundant food supply to lynx [16]. Indeed, only 38% of rabbits supplied were consumed by lynx, and a negligible proportion was taken by other species, which suggest that food provision was *ad libitum* [16]. In A1, food supplementation began in 2002, whereas it was implemented in the other areas since 2006.

**Table 1:** Number of feeding stations (FS) and monitoring effort in four areas of the Doñana Natural Reserve, SW Spain.

Area	(km^2^)	Cumulative number of lynx identified by independent methods	FS	FS with cameras	Effort (camera-nights)	Cumulative number of lynx detected inside FS	Number of lynx records	Composition of the local lynx population^#^					
								Males			Females		
								J	S	A	J	S	A
A1	(40)	**11** (2002–2006)	15	13	42575	**11**	3695	3	3	3	2	1	5
A2	(20)	**10** (2005–2007)	7	7	8967	**10**	1203	2	0	3	2	0	3
A3	(17)	**2** (2006–2007)	3	3	621	**2**	384	0	1	0	0	0	1
A4	(16)	**6** (2006–2007)	2	2	424	**6**	67	1	0	2	2	0	1
Total	93	**28** *	27	25	52587	**28** *	5349	6	4	8	6	1	10

The number and identity of lynx recorded with cameras inside FS matched those identified by independent methods. We show the composition of the lynx population in each area obtained from pictures at FS. Age classes are categorized as juvenile (J; <1 yr), subadult (S, 1–2 yr) and adult (A, >2 yr).

* We identified the same adult male in A1 (November 2002–September 2005) and in A2 (November 2005–March 2007).

# Four individuals used the supplementary food at different age classes (all of them in A1).

We used automatic photographic cameras to identify the individuals that used the supplementary food, and to record the frequency with which each individual visited FS. We placed cameras in 25 out of the 27 FS (Table 1). We used three different systems: 13 Trailmaster® TM1050 active infra-red systems (Goodson Associates Inc., KS, USA) with automatic 32-mm Canon® cameras; 8 Cuddeback® Digital-cameras (Cuddeback® Digital, Non Typical Inc., Park Falls, WI, USA); and 4 Stealth Cam® Digital-cameras (Stealth Cam® LLC, TX, USA). Initially we set two cameras per FS, one in front of each other, until we had pictures of both flanks for all animals detected in a given area; afterwards we used a single camera. Cameras were set at a height of 35 cm. All systems were programmed to run continuously, and to trigger as soon as the infrared beam was intercepted, with a delay of 1 minute between successive pictures. Date and time of each picture were automatically recorded. Cameras were checked once a week.

**Figure 2:**
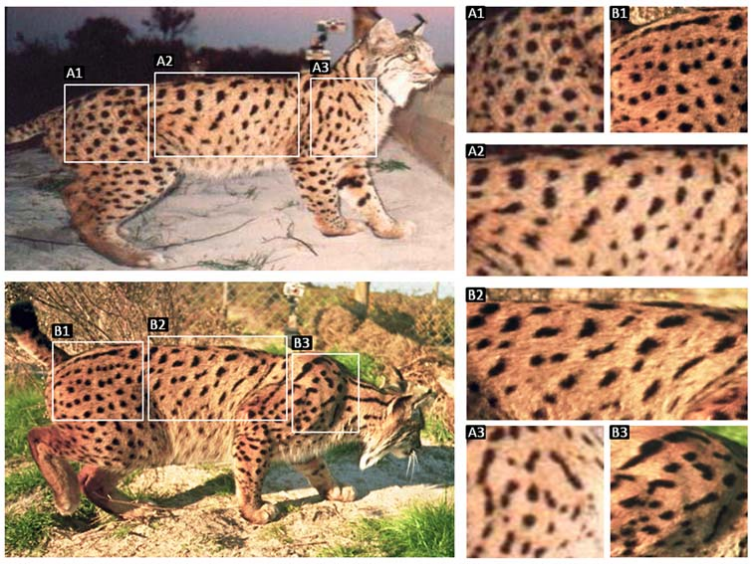
Differences in the pattern (number, position, size and shape) of spots in the flanks of two Iberian lynx. Spot patterns allow unambiguous identification of individuals from photographic records.

We identified individual lynx on pictures from unique patterns in the shape and size of the spots on their flanks (Fig. 2). Spot patterns, sex, age, and reproductive status were known for all lynx in the population, before or after their first detection in FS, using independent methods: intensive trapping and radio-tracking, camera-trapping outside FS, detection of breeding events and subsequent marking of litters, and DNA identification from faecal samples (unpublished data). Yet sex could be sometimes determined in pictures from primary sexual characters, and individuals were easily assigned to an age class from size, morphology and, in the case of juveniles, because they were often photographed together with their mothers.

### Data analyses

Once a new lynx was identified in pictures, we noted whether it was or was not recorded in at least one FS on a daily basis. This binary variable was called daily visit frequency. For each individual we also defined intensity of use as the number of visits to any FS per day. To avoid pseudo-replication we considered that consecutive visits to the same FS were independent if at least 30 minutes elapsed between them, since video recordings showed that the maximum time an individual spent inside a FS was <30 min (n = 71 lynx visits).

**Figure 3:**
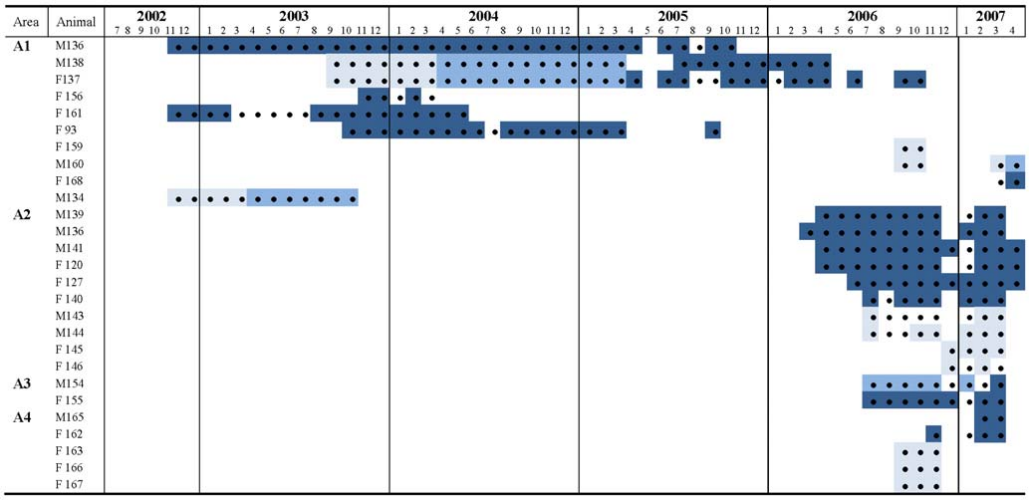
Monitoring of individual lynx in four areas (A1–A4) with feeding stations (FS) within the Doñana region. Blue-shading indicates age class: juvenile (light), subadult (intermediate), and adult (dark). The first blue-shaded cell represents the month when the individual was first detected in a FS. The last blue-shaded cell indicates the month when the lynx died or abandoned the area, or when our study ended (April 2007). Black dots represent camera operation inside FS. Two adult males, one in A1and A4, that were detected only once are not shown.

We assumed that all lynx had free access to every FS available in their home ranges and that the frequency of visits to FS (use) reflected food consumption. These assumptions relied on three facts: a) all resident lynx that made up the local population were recorded in FS (Table 1, Fig. 3), b) 87% of lynx visits to FS ended with the capture of a supplemented rabbit [16], and c) the number of pictures in which lynx had a rabbit in their mouths was similar across sex and age classes (Generalised linear mixed model; sex, F = 0.01, *P = *0.959; age, F = 0.182, *P = *0.165; interaction term, F = 0.29, *P = *0.748; random factors: individual identity and area).

We categorized lynx as juveniles (<1 yr), subadults (1–2 yr), and adults (>2 yr). For the test of the demand hypothesis, we grouped juveniles that did not depend upon their mothers and subadults into an age class called young (<2 yr).

Each year, adult females were classified as breeders if they were observed, or recorded with cameras, in the company of juveniles between July and November; otherwise they were classified as non-breeders that year. Considering the birth date of 30 litters in the study area between 1994 and 2008, most lynx births occurred in March, when females may incur high energetic costs due to lactation. In addition, kittens begin to eat meat at an age of about 30 days [28]. Then, we considered March as the onset of a period of high food demand by breeding females. September was the latest month we ever observed a breeding female feeding their offspring with supplemented rabbits. Therefore, we defined breeding season, in terms of high demand of supplementary food, as the period between March and September. The rest of the year was considered non-breeding season.

Taking into account a gestation period of 65 days and the birth date of the mentioned 30 litters, we determined the pre-mating season and mating season, when we hypothesized that males could demand more supplementary food to enhance their body condition and compete better for territorial females, between November and February, with a peak in late December.

We used body weights of 20 lynx obtained from intensive trapping between October and December 2006 to explore the relationship between body mass and use of supplementary food in a period of 5 months around the date when lynx were caught and weighed.

We quantified three additional factors that could affect consumption of supplementary food in order to control statistically their potential effect. First, spatial and temporal variation in lynx density could produce variability in the strength of competition for food, which in turn could influence the use of supplemental food by individuals of different sex and age classes. Therefore, we used camera records to update lynx density on a monthly basis (Fig. 3). To standardize lynx densities, we estimated the size of the four lynx areas as the minimum convex polygon (MCP) on the pooled positions of radio-tagged lynx per area and month (range 2–10 individuals; unpublished data). MCPs were calculated with the application Home Range for ArcView 3.2 [29]. Second, the availability of FS could affect the use of supplementary food. Then we calculated the daily number of FS available to each individual, which varied across individuals and was dynamic due to the eventual drift of their home ranges. Third, the use of supplementary food is modulated by variations in lynx energetic demands and rabbit abundance [16]. Then, to control this potential effect, we defined three seasons according to lynx behaviour and rabbit abundance: 1) December-March (mating season and medium rabbit abundance), 2) April-July (kitten rearing and high rabbit abundance) and, 3) August-November (females accompanied by juveniles, pre-dispersal phase and low rabbit abundance).

### Statistical methods

To test the hypotheses regarding daily visit frequency and intensity of use, we built generalised linear mixed models (GLMMs) with binomial error and logit link, and Poisson error and log link, respectively, treating individual identity, year and area as random effects. We regressed daily visit frequency and intensity of use against body mass. For these regressions, food utilisation was computed over a five-month period whose central date coincided with our record of lynx weight. We also compared these indices between sexes and age classes for individuals that shared the same breeding territory and had access simultaneously to the same set of FS. In all models we included lynx density, number of FS available, and season in order to control their effects. For a reduced dataset of 10 weeks before and 10 weeks after lynx weight was recorded, we compared the predictive ability of models containing either sex-age classes or body mass as the only predictor using the Akaike's information criterion (AIC), where the model with the lowest AIC value indicates the best model among the examined models [30]. In addition, within the framework of the DDH, we fitted a GLMM with binomial error and logit link to evaluate the interaction between sex, age and the number of FS available on the daily visit frequency. GLMMs were fit using the GLIMMIX macro for the SAS package [31].

## Results

Out of 5349 pictures taken, 2269 events (42%) were independent. We identified 28 lynx inside FS (15 females and 13 males; 18 adults, 5 subadults and 12 juveniles; Table 1, Fig. 3). Two adult non-resident males were photographed only once, so they were not considered for analysis. Males consumed more supplementary food than females, and adults and subadults consumed more supplementary food than juveniles (Table 2). The null hypothesis that the use of supplementary food was homogeneous across sexes and age classes was rejected (Table 2).

**Table 2:** Test of predictions from different hypotheses about ecological mechanisms of asymmetrical use of supplementary food by the Iberian lynx. n = number of lynx.

Hypothesis	Group	n	Daily visit frequency				Intensity of use				
			Mean(±SE)	χ^2^	df	*P*	Mean(±SE)	F	df	*P*	Prediction supported
**1. Null hypothesis**											
1.1. Male = Female	Female	15	0.20±0.03	4.41	1	0.035	1.42±0.09	2.11	1	0.146	No
	Male	11	0.29±0.03				1.52±0.08				
1.2. Adult = Subadult = Juvenile	Adult	16	0.30±0.03	57.91	2	<0.0001	1.58±0.06	3.06	2	0.047	No
	Subadult	5	0.29±0.04				1.55±0.13				
	Juvenile	12	0.15±0.02				1.29±0.09				
**2. Demand hypothesis (DH)**											
2.1 Adult females; breeding season > other seasons	Breeding season	10	0.23±0.06	4.15	1	0.050	1.57±0.09	0.01	1	0.978	No
	Non breeding season	9	0.25±0.04				1.56±0.11				
2.2 Adult males; mating season > other seasons	Mating season	5	0.37±0.04	1.13	1	0.287	1.55±0.18	0.03	1	0.869	No
	Non mating season	5	0.42±0.04				1.54±0.08				
2.3 Breeding females > non-breeding females	Breeding females	6	0.28±0.03	0.67	1	0.413	1.60±0.13	3.04	1	0.090	No
	Non breeding females	5	0.21±0.06				1.53±0.10				
2.4 Young > adult females outside breeding season or adult males outside the mating season	Young females	6	0.11±0.03	16.63	1	<0.0001	1.10±0.10	0.82	1	0.366	No
	Adult females	9	0.24±0.04				1.56±0.11				
	Young males	7	0.25±0.04	4.98	1	0.026	1.44±0.13	0.13	1	0.714	
	Adult males	5	0.42±0.03				1.55±0.08				
**3. Despotic Distribution hypothesis (DDH)**											
3.1 Consumption reflects a hierarchy of status and body size	Adult males	5	0.41±0.03	11.01	2	0.004	1.57±0.08	3.40	1	0.034	Yes
	Adult females	10	0.24±0.04				1.58±0.10				
	Subadult males	4	0.28±0.08				1.56±0.16				
	Subadult females	1	0.32				1.54				
	Juvenile males	7	0.23±0.04				1.50±0.11				
	Juvenile females	6	0.15±0.03				1.10±0.08				

### The demand hypothesis

Tests of the predictions from the demand hypothesis are shown on Table 2. During the breeding season, females did not use more extra food than during the non-breeding seasons. Throughout the pre-mating and mating season, use by adult males did not differ significantly from that during the rest of the year. Breeding females visited FS with higher frequency than non-breeding females but differences were not significant (Table 2). Finally, contrary to the fourth prediction of the demand hypothesis, daily visit frequency by young females was significantly lower than that of adult females outside the breeding season, and it was also lower than that of males outside the mating season. Therefore, none of the four predictions formulated under the DH was supported (Table 2).

### The despotic distribution hypothesis

**Figure 4:**
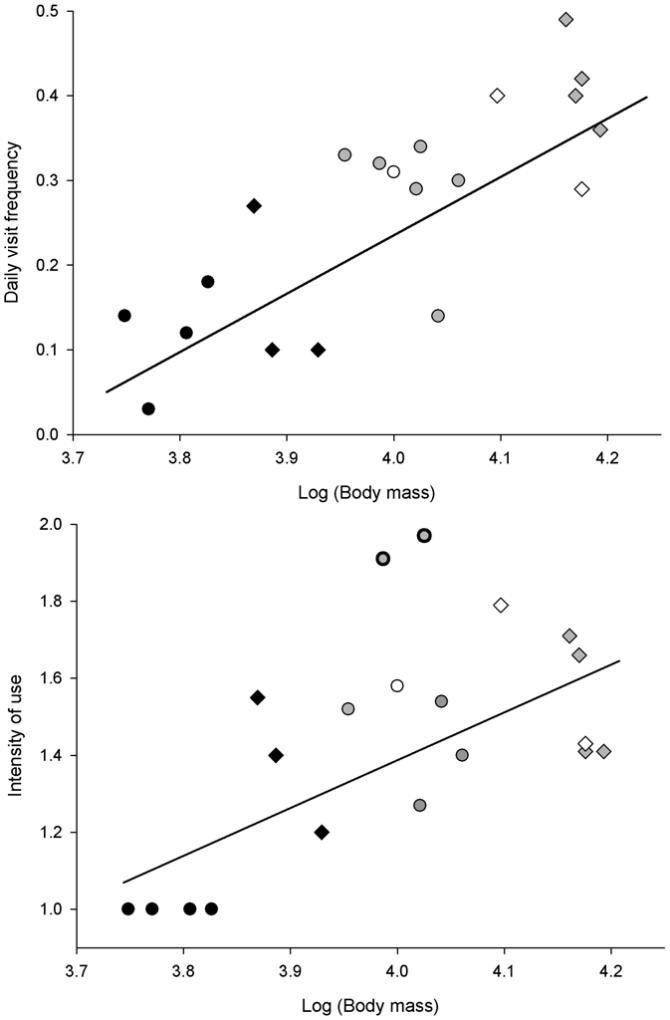
Regression between indices of supplementary food use and Iberian lynx body mass (g). Upper panel: daily visit frequency, R^2^ = 0.655; lower panel, intensity of use, R^2^ = 0.518. Grey diamonds: adult males; grey circles: adult females; white diamonds: subadult males; white circles: subadult female; black diamonds: juvenile males; black circles: juvenile females. We excluded two anomalous adult females (edges marked in bold) in the analysis of intensity of use because one of them did not share her territory with any adult male, and the other exhibited a marked dependence upon supplementary food after being recovered in captivity during February 2006.

**Figure 5:**
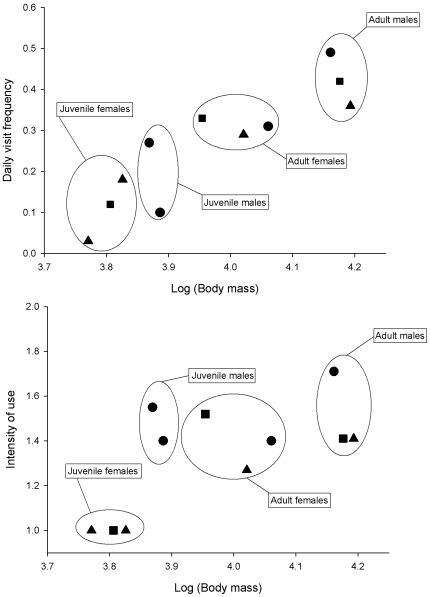
Relationship between indices of supplementary food use and body mass of lynx sharing three breeding territories. Upper panel: daily visit frequency; lower panel: intensity of use. Territory 1: circles; territory 2: triangles; territory 3: squares.

Daily visit frequency in males was higher than in females, and increased with age (Tables 2 and 3). However, adult males, adult females and subadults showed similar intensity of use (median for all groups  = 1 visit/day; Table 2). Adult males used supplementary food with the highest frequency, followed by four sex-age classes with similar mean values of daily visit frequency: adult females, subadults (both sexes), and juvenile males. At the other end, juvenile females exhibited the lowest visit frequency and intensity of use (Table 2). Furthermore, the use of supplementary food increased with body mass (Fig. 4). The effects of hierarchy and body mass held for individuals that shared the same feeding stations in every breeding territory (Fig. 5). Using a five-month reduced dataset, simple models containing only body mass showed a better fit (daily visit frequency: AIC = 4314.6; intensity of use, AIC = 4130.1) than models that included sex-age classes as predictors (daily visit frequency: AIC = 4352.5; intensity of use, AIC = 4182.0).

**Table 3:** Parameter estimates (±SE) in the models that test the null hypothesis of equal use of supplementary food by sexes and age classes in the Iberian lynx.

Model-effect			Daily visit frequency	Intensity of use
			Parameter estimate(±SE)	Parameter estimate(±SE)
Intercept			0.11±0.43	0.44±0.15
Sex		Female	−0.53±0.24*	−0.01±0.07
Age		Juvenile	−0.86±0.23***	−0.01±0.08*
		Subadult	−0.29±0.22	−0.04±0.08
Sex*Age		Female x Juvenile	−0.33±0.33**	−0.33±0.15*
		Female x Subadult	0.60±0.30	0.06±0.12
Covariates				
Season		December-March	0.22±0.08***	0.02±0.04
		April-July	−0.48±0.09	−0.09±0.05
Number of FS			0.05±0.03*	0.02±0.01
Lynx density			−0.03±0.01**	0.01±0.01

The levels “Male”, “Adult” and “Season 3 (August-November)” are included in the intercept.

*Significant at *P*<0.05; ** Significant at *P*<0.01; *** Significant at *P*<0.001.

**Figure 6:**
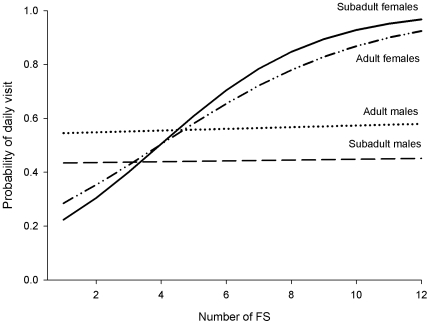
Predicted probability of daily visit for different sex and age classes against the number of available feeding stations (FS).

Depending on the size and location of their home ranges, individual lynx had access to 1–9 feeding stations. The number of FS available within the home range of each individual influenced the use of supplementary food by different sex and age classes. Adult and subadult females tended to use FS at higher rates than adult and subadult males if they had access to at least five FS (*χ^2^* = 5.14, d.f.  = 2, *P = *0.076; Fig. 6).

## Discussion

Identification of lynx in pictures and its comparison with the cumulative number of lynx detected by independent methods demonstrated that all individuals known in the population consumed supplemented rabbits in feeding stations. Steady, high daily visit frequencies also confirmed that all resident lynx, including target individuals such as adult females, made a regular use of supplementary food, as previously suggested [16]. The generalised usage of supplemental food by all individuals may be a direct consequence of the extremely low density of wild rabbits in many sites of the Doñana region [13].

Consumption of supplementary food did not fluctuate with expected temporal variations in the energy needs of different sex and age classes. Therefore, we did not find support for the predictions of the demand hypothesis. Assuming that most lynx visits to feeding stations resulted in consumption [16], supplemented rabbits probably made a substantial fraction of the diet of adult lynx throughout the year. Moreover, food provision was *ad libitum*, that is, in most days we found alive rabbits in feeding stations [16]. These two facts suggest that all individuals could theoretically compensate, or at least attenuate, any potential energy deficit resulting from the scarcity of wild rabbits. In other words, the regular use of food by all individuals in the lynx population suggests that, during the application of the supplementation programme, any discontinuity in reproduction, loss of productivity, or reduced survival could be hardly attributed to food limitation.

Use of supplementary food by the Iberian lynx supported the despotic distribution hypothesis. Although intensity of use was similar across sex and age classes, we found clear asymmetries in the use of feeding stations, particularly in the daily visit frequency: access to feeding stations followed roughly the hierarchy in reproductive status and body size predicted by the DDH (adult males > other sex-age classes > juvenile females). In solitary carnivores, females constitute the most important defendable resource for males [32]. Males need to maximize their body mass and condition to compete for females, exploiting food resources with the lowest cost/benefit ratio whenever possible [33]. Abundance and predictability of supplementary food [16] probably lowers this ratio substantially, as compared with foraging for wild rabbits. Similar asymmetries in the access to food have been reported when strong competition occurs (e.g. food is in short supply) and interactions are often solved through interference [20], [22], [34], [35]. Because of the risk of displacement or aggression by dominants that frequently visit predictable food spots, subordinate individuals, especially juveniles (or adult females accompanied by their offspring) may actively avoid social encounters [34], [36], and may make little use of extra food [37]. Despite lynx did not consume all rabbit provisioned [16]; the density and spatial dispersion of FS may have been low enough to allow dominant adult males to patrol with high frequency all available stations. High chances to find an adult male at a FS may have triggered the inhibition of subordinates, in higher degree for those that rank lowest in the hierarchy. Increasing the number of feeding stations per breeding territory and spreading them as much as possible could make an efficient male patrolling of every enclosure less profitable, thus reducing the probability that subordinates meet dominants. The increment in the daily visit frequency by females and subadults as the number of FS increased is consistent with a relaxation of inhibition in subordinate individuals. Additional support comes from the fact that three subadults made a considerable use of food supplementation in the absence of adult individuals of the same sex. The simplest explanation for the behavioural inhibition of subordinate lynx may be their solitary habits. Outside the mating season, spatiotemporal segregation between adult females, with or without juveniles, and adult males living in the same breeding territory has been reported to be almost complete [26]. However, irrespective of the reduced use of subordinates compared with dominants and its potential causes, in absolute terms all individuals made a sustained, regular use of supplementary food.

Using a reduced dataset (just a five-month period) body mass predicted use of supplemental food better than sex and age classes. This could suggest a partial support of the DH because, all else being equal, heavier individuals have greater energy demands [17]. However, most variation in body mass in the sexually dimorphic Iberian lynx is explained by sex and age [25], and within any given combination of sex and age remarkable differences in use of extra food were observed for animals with equal or very similar weights. Moreover, in the absence of adult males, intensity of use by some adult females was much higher than expected from the relationship predicted by body mass alone (Fig. 4). Therefore, we interpret that the positive effect of body mass basically supports the DDH.

A continuous, regular consumption by most individuals suggests that extra food helped (it could have been crucial in some cases) to retain lynx in areas almost deprived of wild rabbits. More generally, supplementary food may be useful to retain the bulk of a lynx population during extended periods of natural food shortage. Active management of wild rabbit populations has been performed chiefly within the Doñana reserve [38], with limited results, at least in the short term [13]. Since survival of adult and dispersing lynx within the protected area is much higher than outside it [14], lynx retention in the protected area by means of supplementary food may help to maximize the viability of the Doñana population [39] and, since only two lynx populations remain in the wild [40], the preservation of this endangered species.

However, we note that, if the total amount of supplemental food is lower, more clumped, or supplied with lower frequency, or lower spatial predictability than in our supplementation protocol [16], a higher intensity of FS use by competitively dominant adult males may occur. A similar trend towards monopolization of extra food by a fraction of the population has been reported in other species [22], [34], [41]. Competitive asymmetries, in turn, may result in a more pronounced inhibition of juveniles, or even adult females, which at some point would seriously compromise the efficiency of the supplementary feeding programme for such target sex and age classes. For the Iberian lynx, we propose that at least five feeding stations per breeding territory could ensure a regular use by all individuals, and potential inhibition of subordinates could be neutralized.

We emphasize the importance of understanding the role that individual attributes play in wildlife management plans [42], [43]. Individual variation in the use of food may affect the success of supplementary feeding programmes. Finding thresholds in the amount of food that should be provided to satisfy the needs of target individuals could be valuable in species where intraspecific interference seems more likely [22], [34].

## References

[1] Murray DL (2002). Differential body condition and vulnerability to predation in snowshoe hares.. J Anim Ecol.

[2] Preston KL, Rotenberry JT (2006). Independent effects of food and predator mediated processes on annual fecundity in a songbird.. Ecology.

[3] Boland CRJ, Heinsohn R, Cockburn A (1997). Experimental manipulation of brood reduction and parental care in cooperatively breeding white-winged choughs.. J Anim Ecol.

[4] Nagy LR, Holmes RT (2005). Food limits annual fecundity of a migratory songbird: an experimental study.. Ecology.

[5] Partridge ST, Nolte DL, Ziegltrum GJ, Robbins CT (2001). Impacts of supplemental feeding on the nutritional ecology of black bears.. J Wildl Manage.

[6] Phillips MK, Parker WT (1988). Red wolf recovery: a progress report.. Conserv Biol.

[7] Elliot GP, Merton DV, Jansen PW (2001). Intensive management of a critically endangered species: the kakapo.. Biol Cons.

[8] López-Bao JV, Palomares F, Rodríguez A, Delibes M (2009). Effects of food supplementation on home range size, productivity and recruitment in a small population of Iberian lynx.. Anim Cons. In press.

[9] Schoech SJ, Bridge ES, Boughton RK, Reynolds SJ, Atwell JA (2008). Food supplementation: A tool to increase reproductive output? A case study in the threatened Florida scrub-jay.. Biol Cons.

[10] Delibes M, Rodríguez A, Ferreras P (2000). Action plan for the conservation of the Iberian lynx (*Lynx pardinus*) in Europe. Nature and Environment Series, 111.. Council of Europe Publishing, Strasbourg, France.

[11] Rodríguez A, Delibes M (2002). Internal structure and patterns of contraction in the geographic range of the Iberian lynx.. Ecography.

[12] Palomares F, Delibes M, Ferreras P, Fedriani JM, Calzada J (2001). Spatial ecology of Iberian lynx and abundance of European rabbits in southwestern Spain.. Wildl Monogr.

[13] Moreno S, Beltrán JF, Cotilla I, Kuffner B, Laffitte R (2007). Long-term decline of the European wild rabbit (*Oryctolagus cuniculus*) in south-western Spain.. Wildl Res.

[14] Ferreras P, Delibes M, Palomares F, Fedriani JM, Calzada J (2004). Proximate and ultimate causes of dispersal in the Iberian lynx (*Lynx pardinus*).. Behav Ecol.

[15] Palomares F, Revilla E, Calzada J, Fernández N, Delibes M (2005). Reproduction and pre-dispersal survival of Iberian lynx in a subpopulation of the Doñana National Park.. Biol Cons.

[16] López-Bao JV, Rodríguez A, Palomares F (2008). Behavioural response of a trophic specialist, the Iberian lynx, to supplementary food: patterns of food use and implications for conservation.. Biol Cons.

[17] Aldama JJ, Beltrán JF, Delibes M (1993). Energy expenditure and prey requirements of free-ranging Iberian lynx in southwestern Spain.. J Wildl Manage.

[18] Gittleman JL, Thompson SD (1988). Energy allocation in mammalian reproduction.. Amer Zool.

[19] Randall B, Marilyn D, Ronald L (1987). Foraging efficiencies and techniques of juvenile and adult northern mockingbirds (*Mimus polyglottos*).. Behaviour.

[20] Donázar JA, Travaini A, Ceballos O, Rodríguez A, Delibes M (1999). Effects of sex-associated competitive asymmetries on foraging group structure and despotic distribution in Andean condors.. Behav Ecol Sociobiol.

[21] Rode KD, Farley SD, Robbins CT (2006). Sexual dimorphism, reproductive strategy, and human activities determine resource use by brown bears.. Ecology.

[22] Bosè M, Sarrazin F (2007). Competitive behaviour and feeding rate in a reintroduced population of Griffon vultures (*Gyps fulvus*).. Ibis.

[23] Parker GA, Sutherland WJ (1986). Ideal free distributions when individuals differ in competitive ability: phenotype-limited ideal free models.. Anim Behav.

[24] Lewis RJ (2002). Beyond dominance: The importance of leverage.. Q Rev Biol.

[25] Beltrán JF, Delibes M (1993). Physical characteristics of Iberian lynxes (*Lynx pardinus*) from Doñana, southwestern Spain.. J Mammal.

[26] Ferreras P, Beltrán JF, Aldama JJ, Delibes M (1997). Spatial organization and land tenure system of the endangered Iberian lynx (*Lynx pardinus*, Temminck, 1824).. J Zool Lond.

[27] Palomares F (2001). Vegetation structure and prey abundance requirements of the Iberian lynx: implications for the design of reserves and corridors.. J Appl Ecol.

[28] Fernández N, Palomares F, Delibes M (2002). The use of breeding dens and kitten development in the Iberian lynx (*Lynx pardinus*).. J Zool Lond.

[29] Rodgers AR, Carr AP (1998). HRE: The Home Range extension for ArcView. Ontario Ministry of Natural Resources, Centre for Northern Forest Ecosystem Research, Thunder Bay, Ontario, Canada..

[30] Burnham KP, Anderson DR

[31] Littell RC, Milliken GA, Stroup WW, Wolfinger RD (1996). SAS system for mixed models.. SAS Institute Inc, Cary, NC.

[32] Sandell M, Gittleman JL (1989). The mating tactics and spacing patterns of solitary carnivores..

[33] Funston PJ, Mills MGL, Biggs HC, Richardson PRK (1998). Hunting by male lions: ecological influences and socio-ecological implications.. Anim Behav.

[34] Meretsky VJ, Mannan RW (1999). Supplemental feeding regimes for Egyptian vultures in the Negev Desert, Israel.. J Wildl Manage.

[35] Ruckstuhl KE, Neuhaus P (2005). Sexual segregation in vertebrates: ecology of the two sexes..

[36] Bekoff M (1977). Mammalian dispersal and the ontogeny of individual behavioural phenotypes.. Am Nat.

[37] Galindo-Leal C, Krebs CJ (1998). Effects of food abundance on individual and populations of the rock mouse *(Peromyscus difficilis)*.. J Mamm.

[38] Moreno S, Villafuerte R (1995). Traditional management of scrubland for the conservation of rabbits *Oryctolagus cuniculus* and their predators in Doñana National Park, Spain.. Biol Cons.

[39] Revilla E, Rodríguez A, Román J, Palomares F, Ramírez L, Asensio B (2007). Análisis de la viabilidad de la metapoblación de lince ibérico de Doñana: una estrategia de manejo adaptativo para su conservación..

[40] Ferreras P, Rodríguez A, Palomares F, Delibes M, Macdonald DW, Loveridge AJ (2009). Iberian lynx: The uncertain future of a critically endangered cat..

[41] Monaghan P, Metcalfe NB (1985). Group foraging in wild brown hares: effects of resource distribution and social status.. Anim Behav.

[42] Gordon IJ, Hester AJ, Festa-Bianchet M (2004). The management of wild large herbivores to meet economic, conservation, and environmental objectives.. J Appl Ecol.

[43] Gosling LM, Sutherland WJ (2000). Behaviour and Conservation..

